# Supplementary figures and images for: Metabolic modeling predicts metabolite changes in Mycobacterium tuberculosis
Source: BMC Syst Biol. 2015 Sep 16;9:57. doi: 10.1186/s12918-015-0206-7 (PMC4574064; doi:10.1186/s12918-015-0206-7)

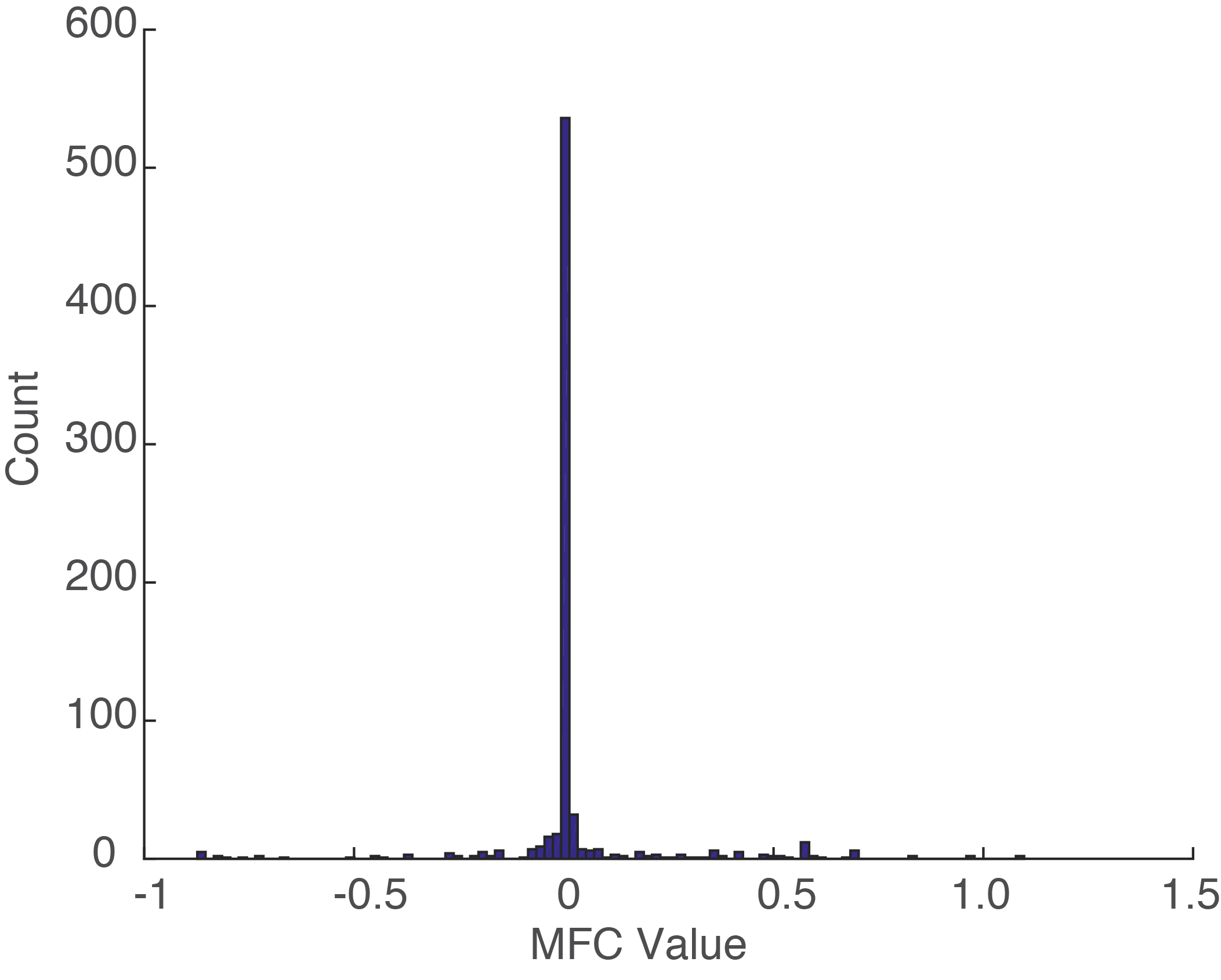

Supplement: Additional file 1: Figure S1. — Distribution of maximum flux capacities for all model metabolites. Histogram of MFC values for all model metabolites. Forty percent of the metabolites in our model (305/754) have an MFC of zero. Three-hundred of these are neither produced nor consumed in our model, likely due to medium constraints placed on the model. External hydrogen is not plotted due its large MFC (approximately 48.4). (PNG 39 kb) [file 12918_2015_206_MOESM1_ESM.png]

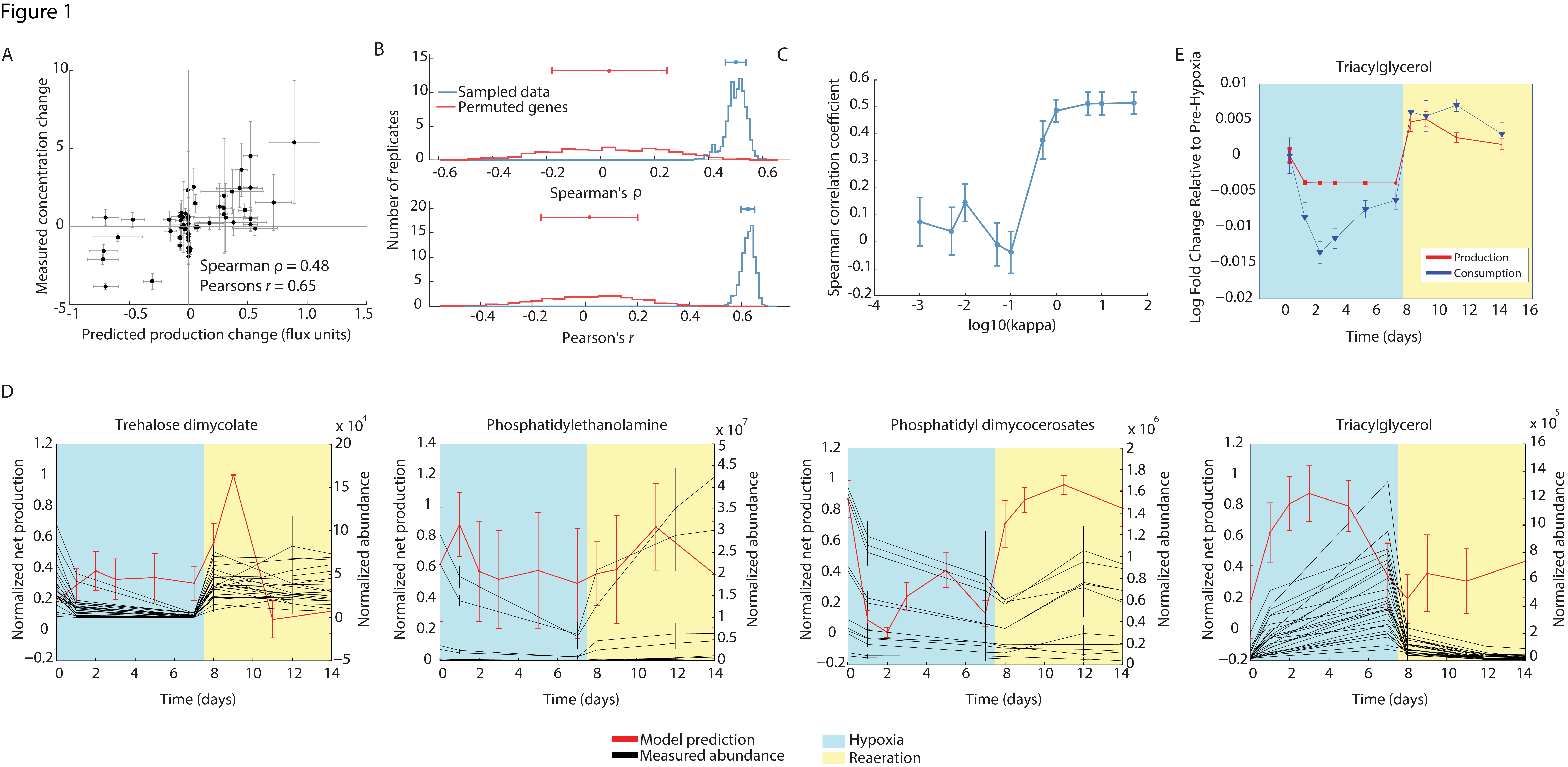

Supplement: Additional file 2: — Supplementary data files. Pathways.zip full set model predictions for all metabolites during transcription factor induction based on genome-wise expression. Pathways_specific.zip full set model predictions for all metabolites during transcription factor induction based on TF regulon specific expression. Model.xml: Genome scale MTB metabolic model used. Table S1 the binding network used for the transcription factor overexpression analyses and median-scaled metabolite abundance values for normoxic and hypoxic conditions. (ZIP 2706 kb) [file 12918_2015_206_MOESM2_ESM.zip › Figure 1.png]

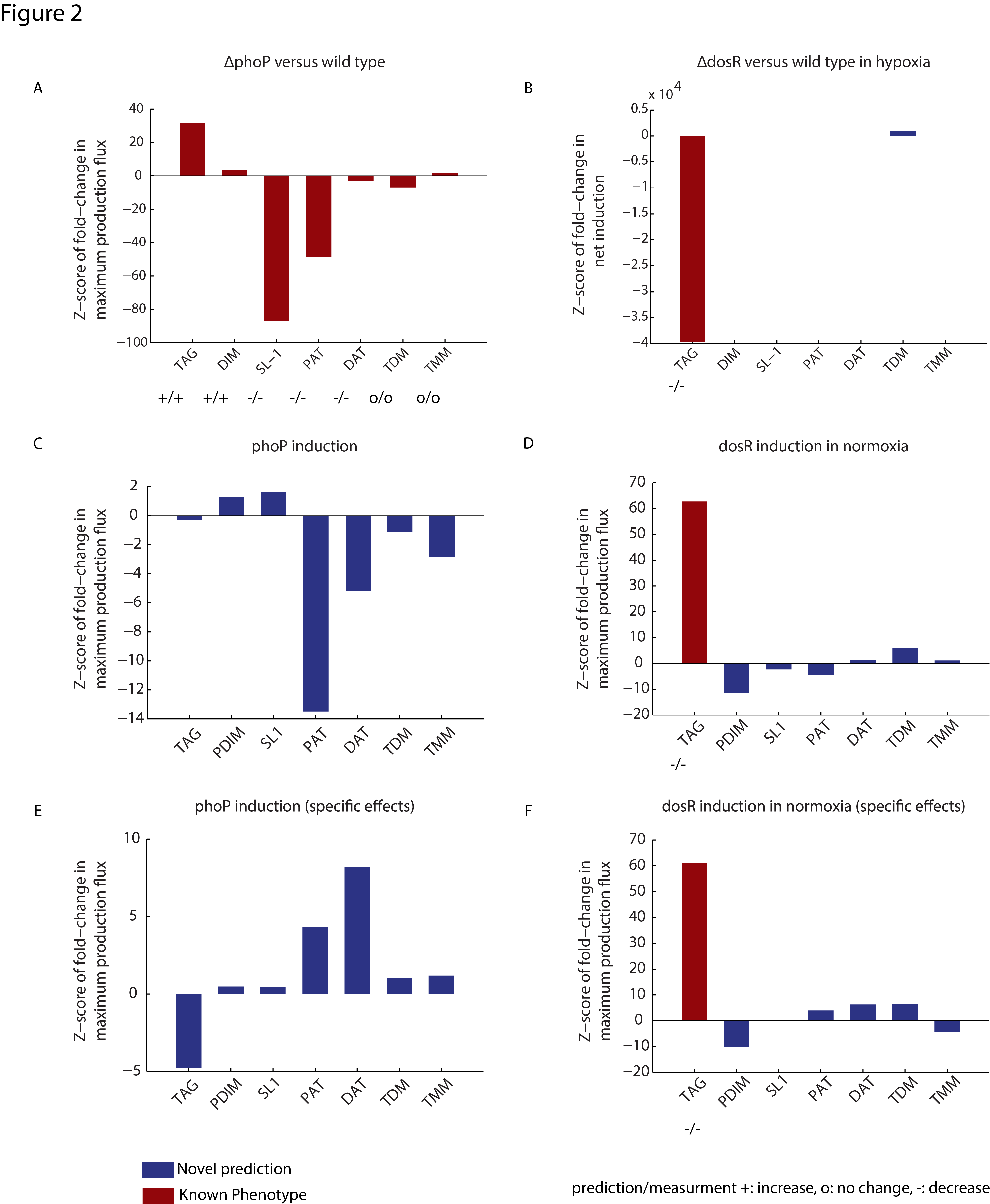

Supplement: Additional file 2: — Supplementary data files. Pathways.zip full set model predictions for all metabolites during transcription factor induction based on genome-wise expression. Pathways_specific.zip full set model predictions for all metabolites during transcription factor induction based on TF regulon specific expression. Model.xml: Genome scale MTB metabolic model used. Table S1 the binding network used for the transcription factor overexpression analyses and median-scaled metabolite abundance values for normoxic and hypoxic conditions. (ZIP 2706 kb) [file 12918_2015_206_MOESM2_ESM.zip › Figure 2.png]
