# Supplementary material for: Metabolic modeling predicts metabolite changes in Mycobacterium tuberculosis
Source: BMC Syst Biol. 2015 Sep 16;9:57. doi: 10.1186/s12918-015-0206-7 (PMC4574064; doi:10.1186/s12918-015-0206-7)
Supplement: Additional file 2: — Supplementary data files. Pathways.zip full set model predictions for all metabolites during transcription factor induction based on genome-wise expression. Pathways_specific.zip full set model predictions for all metabolites during transcription factor induction based on TF regulon specific expression. Model.xml: Genome scale MTB metabolic model used. Table S1 the binding network used for the transcription factor overexpression analyses and median-scaled metabolite abundance values for normoxic and hypoxic conditions. (ZIP 2706 kb) [file 12918_2015_206_MOESM2_ESM.zip › Garay et al BMC Sysbio Final.docx]

# Metabolic modeling predicts metabolite changes in *Mycobacterium tuberculosis*

Christopher D Garay1*, Jonathan M Dreyfuss1,2, James E Galagan1,3,4#

1. Boston University, Department of Biomedical Engineering, Boston, MA 02215, USA

2. Joslin Diabetes Center, Boston, MA 02215, MA

3. Boston University, Graduate Program in Bioinformatics, Boston, MA 02215, USA

4. National Emerging Infectious Diseases Laboratories, Boston, MA 02118, USA

# To whom correspondence should be addressed: [jgalag@bu.edu](mailto:jgalag@bu.edu)

Email: Christopher Garay: [cgaray@bu.edu](mailto:cgaray@bu.edu), Jonathan Dreyfuss: [jdreyf@bu.edu](mailto:jdreyf@bu.edu), James Galagan: jgalag@bu.edu

Running title: Modeling metabolism in *Mycobacterium tuberculosis*

# Abstract

**Background**: *Mycobacterium tuberculosis* (MTB) is the causal agent of the disease tuberculosis (TB). Metabolic adaptations are thought to be critical to the survival of MTB during pathogenesis. Computational tools that can be used to study MTB metabolism *in silico* and prioritize resource-intensive experimental work could significantly accelerate research.

**Results**: We have developed E-Flux-MFC, an enhancement of our original E-Flux method that enables the prediction of changes in the production of external and internal metabolites corresponding to gene expression measurements. We have used this method to simulate the changes in the metabolic state of *Mycobacterium tuberculosis* (MTB). We have validated the accuracy of E-Flux-MFC for predicting changes in lipids and metabolites during a hypoxia time course using previously published metabolomics and transcriptomics data. We have further validated the accuracy of the method for predicting changes in MTB lipids following the deletion and induction of two well-studied transcription factors (TFs). We have applied the method to predict the metabolic impact of the induction of each of the approximately 180 MTB TFs using a previously generated and publically available expression data set.

**Conclusions**: E-flux-MFC can be used to study global changes in MTB metabolites from gene expression data associated with environmental and genetic perturbations. The application of this method to a data set of MTB TF perturbations provides a resource for studying the large number of TFs whose functions remain unknown. Most TFs impact metabolites indirectly through the propagation of gene expression changes through the regulatory network rather than through their direct regulons. E-Flux-MFC is also applicable to any organism for which accurate metabolic models are available.

**Keywords:** flux balance analysis, *Mycobacterium tuberculosis*, genome-scale metabolic modeling, metabolomics, transcription factor regulation

# Introduction

*Mycobacterium tuberculosis* (MTB) is the causal agent of the disease tuberculosis (TB). With more than 9 million new cases of active disease and nearly 1.5 million deaths in 2013, TB is a global health emergency of substantial proportions ([1](#_ENREF_1)). This is amplified by the emergence of mono-resistant, multiple drug resistant (MDR), extensively drug resistant (XDR) and, most recently, totally drug resistant (TDR) strains of TB ([2-6](#_ENREF_2)). MTB is primarily transmitted to a new host via inhalation ([7](#_ENREF_7)). Within the lung MTB is phagocytosed by macrophages, which ultimately triggers the formation of a granuloma that contains the infected cells. The success of this microbe is due in part to its ability to survive within the granuloma for long periods of time (sometimes decades) in an asymptomatic state ([8-10](#_ENREF_8)). One-third of the world’s population is latently infected with MTB ([11](#_ENREF_11)).

Metabolic adaptations are thought to be critical to the survival of MTB during pathogenesis. Within the host, the bacterium must adapt to a range of stresses including hypoxic ([12-24](#_ENREF_12)), acidic ([25](#_ENREF_25)), nitrosative ([26](#_ENREF_26)), and redox ([27-30](#_ENREF_27))stresses. The response of MTB to hypoxia in particular is characterized by widespread metabolic changes including the induction of cholesterol catabolism (even independent of the presence of cholesterol), alterations in the metabolism of triacylglycerides (TAG), alterations in methyl-branched lipids, the rapid release of free mycolates from trehalose dimycolates (TDM), and widespread changes in both intracellular and extracellular amino acid levels ([31](#_ENREF_31)). Restrictions in nutrient availability further characterize the host environment. Within the host, MTB shifts to lipids ([32](#_ENREF_32), [33](#_ENREF_33)) and host-derived cholesterol ([34-39](#_ENREF_34)) as primary nutrient sources. In addition, several lines of evidence indicate that an MTB infection results in metabolic reprogramming of the macrophage host ([9](#_ENREF_9), [40](#_ENREF_40), [41](#_ENREF_41)). Although the roles of a small number of transcriptional regulators have been well studied in connection to these adaptations ([23](#_ENREF_23), [25](#_ENREF_25), [29](#_ENREF_29), [30](#_ENREF_30)), the regulatory mechanisms underlying these changes remain largely unknown. In fact, the potential regulatory roles of the vast majority of the approximately 180 predicted transcription factors of MTB remain unknown.

Experimental work with MTB is challenging and resource intensive. Work must be performed in a specialized BSL-3 environment, and fewer genetic tools exist for MTB relative to other organisms. Experimental work is also burdened by the extremely slow growth rate of the organism. MTB has a doubling time of approximately 24 hours (as compared to 20 minutes for *E. coli*). Experiments with MTB thus require months to perform. Computational tools that can be used to study MTB in silico and prioritize resource-intensive experimental work could thus significantly accelerate research.

Computational metabolic modeling has been applied successfully to gain insight into the metabolism of MTB ([42-49](#_ENREF_42)). These efforts are complemented by a large number of computational studies into the metabolism of other non-pathogenic and pathogenic bacterial strains ([50-54](#_ENREF_50)) as well as human metabolism ([55](#_ENREF_55), [56](#_ENREF_56)). Two widely used models of MTB metabolism have been published: iNJ661 ([42](#_ENREF_42)) and GSMN-TB ([43](#_ENREF_43), [57](#_ENREF_57)). Further enhancements to these models have addressed shortcomings in the original model by incorporating new biochemical knowledge collected about key pathways in mycobacterial metabolism, allowing researchers to develop metabolic models specific to conditions that are important for understanding the processes that underlie virulence ([47](#_ENREF_47), [57](#_ENREF_57), [58](#_ENREF_58)).

Genome-scale metabolic models capture information about both experimentally validated and computationally predicted biochemical processes within an organism in the form of a stoichiometric matrix. These models also describe the relationship between genes, proteins, and the enzymes that catalyze each reaction in the organism ([59](#_ENREF_59)). Flux balance analysis (FBA) is a method that is used to predict network metabolic capabilities at steady state ([60-64](#_ENREF_60)). The main assumption of standard FBA is the absence of the production or consumption of metabolites, outside of select source or sink metabolites describing nutrient uptake, waste secretion and biomass production ([59](#_ENREF_59)). Traditional implementations of FBA typically do not include terms that describe kinetic parameters, feedback inhibition, or the effects of transcriptional regulation on reaction fluxes. FBA has been used successfully to predict the metabolic phenotype of gene knockouts ([65-67](#_ENREF_65)), to predict growth rates ([43](#_ENREF_43), [51](#_ENREF_51), [57](#_ENREF_57), [68-70](#_ENREF_68)), and to predict rates of metabolite uptake and secretion across time using quasi-steady-state modeling approaches ([44](#_ENREF_44), [46](#_ENREF_46), [71](#_ENREF_71), [72](#_ENREF_72)).

Many methods have been developed to couple gene expression state with FBA and have been reviewed in depth by Lewis et al. ([73](#_ENREF_73)). One of the first of these methods, called rFBA, utilized Boolean constraints in order to simulate changes in metabolic flux in response to changes in environmental conditions and regulatory network perturbations ([74](#_ENREF_74)). Other methods, such as GIMME and iMAT, utilize gene expression microarray data in order to generate flux solutions that are consistent with a set of gene expression data ([50](#_ENREF_50), [75](#_ENREF_75), [76](#_ENREF_76)). Previously, we described a method called E-Flux that extends FBA by translating gene expression data into hard constraints on the maximum metabolic flux through individual reactions ([46](#_ENREF_46), [68](#_ENREF_68)). Using E-Flux, we predicted the impact of drugs on MTB mycolic acid biosynthesis ([46](#_ENREF_46)). By integrating expression data from a compendium of 437 microarray experiments corresponding to 75 different drugs and culture conditions ([77](#_ENREF_77)), we correctly predicted 6 of 7 known inhibitors within this data set ([46](#_ENREF_46)). A more recent method, called Probabilistic Regulation of Metabolism (PROM) ([49](#_ENREF_49)), translates expression data into soft constraints on model reaction rates. PROM generates a flux distribution that is consistent with a set of gene expression data by minimizing the sum of the violations on these constraints across all of the reactions in the model. E-Flux is tailored to predicting terminal, or sink, metabolites and the PROM method is easily adaptable for the study of those same metabolites.

Here, we present E-Flux-MFC, an enhancement of the original E-Flux method that enables the accurate prediction of changes in the production of both external and internal metabolites by integrating gene expression data. We have validated the accuracy of E-Flux-MFC in predicting changes in lipids and metabolites during a hypoxia time course using previously published metabolomics and transcriptomics data ([31](#_ENREF_31)). We have further validated the accuracy of E-Flux-MFC for predicting changes in MTB lipids following the deletion and induction of two well-studied transcription factors. We then use our validated approach to predict the metabolic impact of the induction of each MTB TF using a previously generated and publicly available expression data set ([31](#_ENREF_31), [78](#_ENREF_78)). These predictions provide a resource for studying the large number of TFs whose functions remain unknown, and for identifying TFs that may play direct or indirect role in regulating the metabolism of compounds of interest.

# Results

## Metabolic Modeling with E-Flux-MFC

Traditionally, FBA approaches have relied on the steady-state assumption, which constrains the total change in concentration of each metabolite to some constant value. Recent approaches have addressed the problem of modifying FBA in order to make predictions of changes in the consumption or production of model metabolites ([71](#_ENREF_71), [72](#_ENREF_72), [79](#_ENREF_79)). Here we have developed a method to predict changes in the accumulation and utilization of both extracellular and intracellular metabolites by relaxing the steady-state constraint for each of the metabolites in our model and then calculating the difference between the maximum production flux and the maximum consumption flux in order to calculate a value that we call maximum flux capacity (MFC).

In order to validate our method, we examined several published gene expression data sets associated with experimentally measured changes in metabolite production. One data set comprises both gene expression data and metabolomics data for 69 metabolites in GSMN-TB. These data were collected during a time course experiment in which *Mycobacterium tuberculosis* H37Rv was exposed to hypoxia ([31](#_ENREF_31)). This data set thus combines gene expression and metabolite measurements in conditions relevant to TB pathogenesis. Two additional data sets are expression datasets associated with knockouts of the lipid-production associated transcription factors *phoP* (Rv0757) ([25](#_ENREF_25)) and *dosR* (Rv3133c) ([23](#_ENREF_23)). These are the only two TF deletion studies in MTB, of which we are aware, that have coupled both transcriptomics and metabolomics. These data were used to validate the accuracy of our approach in predicting the metabolic impacts of TF deletions.

Importantly, because our method is an adaptation of FBA, our model generates predictions of metabolite production or secretion at a quasi-steady-state that is defined by both the medium constraints placed on the model and the gene expression data from a particular time point. Our predictions are not predictions of changes in concentration over time (which would rely on precise measurements of initial metabolite measurements and medium uptake and secretion rates), but are instead qualitative predictions of changes in maximum production. We compare these predictions against measured changes in concentration. We propose that decreases and increases in maximum flux capacity generally lead to corresponding decreases and increases in metabolite concentration respectively.

### Prediction of changes in metabolite production in a hypoxic time course

As a first validation of our approach, we sought to predict changes in lipid production in response to exposure to hypoxia, which generates a complex regulatory response that allows MTB to survive within a low-oxygen environment. In previously published work, MTBwas subjected to a time course of hypoxia during which the relative levels of transcripts, metabolites, and selected lipids were measured ([31](#_ENREF_31)). These data sets provide a systems-level compendium of experimental data that describes MTB’s response to a trigger for entry into dormancy.

For our method we utilized gene expression data collected across a hypoxic time course in order to generate reaction bounds. In order to model the uncertainty in our gene expression values and their relationship to modeling predictions, we utilized a Monte Carlo sampling approach. For each gene at each time point we added values sampled from a Gaussian distribution centered on zero with a standard deviation calculated based on replicate measurements. These samples were added to the log2 RMA expression values and subsequently exponentiated for reaction expression calculation. Similar approaches have been used previously in order to assess the sensitivity of modeling results on the variance of gene expression data ([46](#_ENREF_46), [68](#_ENREF_68)).

In Figure **1**A, we show the results for a comparison between 24 hours after the introduction of hypoxia and pre-hypoxic conditions. We compare log-fold changes in maximum flux capacity with log-fold changes in metabolite abundance for each metabolite that was measured in this experiment and that was also present in the MTB metabolic model (Figure S1 provides a histogram of MFC values for all metabolites in our model). In order to assess the relationship between changes in MFC and changes in concentration, we calculated the Spearman correlation coefficient. For the hypoxic transition data set, we calculate a value of 0.48 (p=1.7x10-5). Although we do not necessarily expect a linear relationship between MFC and change in metabolite abundance with our method, we also calculate a Pearson correlation coefficient of 0.65 (p=1.1x10-9). Even in the absence of detailed kinetic parameters for each reaction and the lack of quantitative concentration measurements, our predictions are positively correlated with changes in concentration after the induction of hypoxia. Importantly, our predictions encompass both intracellular and extracellular metabolites.

Alterations of oxygen tension results in the down-regulation of the majority MTB genes coupled with the up-regulation of a smaller subset ([31](#_ENREF_31)). In order to assess whether the correlation between our predictions and changes in metabolite concentration was due to these broad patterns of expression changes versus the modulation of specific genes, we performed a permutation analysis designed to assess the specificity of our results (Figure **1**B). Similar methods have been described previously ([46](#_ENREF_46), [54](#_ENREF_54), [68](#_ENREF_68)). We performed 1000 permutations by randomizing gene labels then applying the resulting expression set to our model. We compared the distribution of Spearman correlation coefficients for the correct gene labels with predictions generated after randomizing gene labels. Our model performs significantly better with data from the true distribution, suggesting that our predictions are due to the specific configuration of gene expression and the metabolites that we are testing and not due to a global trend in the expression data.

In addition to random sampling and gene label permutation analysis, we examined the effect of the parameter (see ). controls the hardness of the expression-based reaction bounds. As approaches 0, these constraints become much softer and have less of an effect on the final solution. As increases, the expression-based reaction bounds become much harder bounds and thus place a much greater constraint on the model ([49](#_ENREF_49)). Higher values of generate bounds that are similar to the hard reaction constraints imposed by E-Flux. For each value of , we generated 100 samples of our hypoxic transition gene expression data. Figure **1**C shows the results of this analysis. We have plotted both the mean and standard deviation of the Spearman correlation coefficient for the MFC values that we calculate from these samples for each value of the parameter. Our sensitivity analysis suggests that past values of approximately 1.0, there is little variation in the Spearman correlation coefficient. Similarly to Chandrasekaran and Price ([49](#_ENREF_49)), we find that a value for of 1.0 strikes a balance between hard and soft bounds that yields reasonable predictions for our analysis of both hypoxic transition data and transcription factor knockout data.

We further predicted the impact of hypoxia on the production of key mycobacterial lipids and compared these changes to measured changes in lipids ([31](#_ENREF_31)). In Figure 1D we show comparisons between predictions (in red) and measurements (in black) for several classes of lipids during both hypoxia (blue) and subsequent reaeration (yellow). For visualization, production values are normalized by first subtracting the minimum MFC value for each species across time, then dividing by the difference between the maximum and the minimum MFC values. The measurements of changes in lipids are plotted for individual molecular species belonging to broad classes of lipids. Our results demonstrate qualitative agreement between our predictions of lipid maximum net production rates and measured changes in lipid levels. Assuming no other processes alter lipid levels, changes in lipid amounts should reflect the time integral of net production. Qualitatively, this is what we observe. Decreases in trehalose dimycolates (TDMs) and phosphatidyl dimycocerosates (PDIMs) during hypoxia and increases during reaeration are correlated with decreases and increases in predicted net production, respectively. Similarly, increases in triacylglycerides (TAGs) during hypoxia and decreases during re-aeration are correlated with increases and decreases in predicted net production, respectively. Predictions for phosphatidylethanolamine (PE) have substantially higher variance and we do not predict significant changes in the production of PEs at either transition. This is consistent with measured changes in abundance for some members of this class, but not others.

MTB is known to accumulate TAG during hypoxia and utilize TAG during subsequent re-aeration ([80-83](#_ENREF_80)). The consumption of TAG is necessary for the reactivation of non-replicating *Mycobacterium bovis* bacillus Calmette-Guérin (BCG) after exposure to hypoxia ([82](#_ENREF_82)). TAGs also play a role in maintaining redox homeostasis by acting as a source of reducing equivalents ([29](#_ENREF_29)) and the modulation of carbon flux away from the TCA cycle and into the production of TAG (which leads to slowed growth) has been shown to be an important modulator of the response of MTB to treatment by antibiotics ([84](#_ENREF_84)). In principle, the accumulation of TAG during hypoxia might be driven by an increase in production, a decrease in consumption, or both. We examined this by separately predicting the maximum production and consumption of TAG over the time course (Figure 2E). The results suggest that the increase in TAG during hypoxia is driven largely by a decrease in consumption relative to production. This is consistent with the overall decrease in growth during hypoxia-induced dormancy. Moreover, of the 24 MTB lipase genes predicted to cleave acyl groups from TAG ([81](#_ENREF_81)), 18 show repression during hypoxia ([31](#_ENREF_31)). Conversely, during re-aeration, both consumption and production increase, but greater relative consumption is predicted to drive lower TAG abundance. Consistent with this, during re-aeration the expression of most lipase genes returns to baseline, and genes involved in fatty acid synthase I (FAS1), energy metabolism and β-oxidation show increased expression ([31](#_ENREF_31)).

### Comparison of E-flux-MFC with E-flux and PROM

As noted above, E-flux-MFC is an extension of E-flux. E-flux calculates the maximum production of sink metabolites and is thus tailored for external metabolites that are the product of unidirectional reactions. E-flux-MFC extends E-flux to internal metabolites by predicting both the maximum production and consumption of a metabolite to calculate the MFC. To assess the increase in accuracy attained by this enhancement, we evaluated the prediction of standard E-flux on the hypoxia data set by calculating only the maximum production of all metabolites. For this analysis, the Spearman correlation coefficient was 0.16 (p=0.09) and the Pearson correlation coefficients was 0.30 (p=0.01). As expected, E-flux-MFC performs considerably better than E-flux.

E-Flux-MFC also borrows from PROM method, which implements soft reaction bounds. PROM predicts biomass production rather than changes in the production of individual metabolites. However, PROM can be used in the same manner as E-flux by treating each metabolite as a single element biomass vector. We implemented PROM in this way to compare with E-flux-MFC on the hypoxia data set. For this analysis, the Spearman correlation coefficient was 0.36 (p=0.001) and the Pearson correlation coefficient was 0.35 (p=0.002). E-flux-MFC thus also performed better than PROM, although PROM performed better than standard E-flux.

### Predicting changes in lipid expression after *phoP* or *dosR* deletions.

Our initial validation suggests that our approach is capable of predicting qualitative changes in metabolite levels based on gene expression changes from wild-type cells. Toward the goal of supporting experimental design, we also sought to test the ability of our approach to predict metabolic changes corresponding to gene deletions.

We first analyzed the knockout of the transcription factor PhoP ([25](#_ENREF_25)) (GSE22854). This study compared the transcriptional responses of the CDC1551 strain of MTB to that of a *phoP* transposon mutant. Each strain was grown to early log phase in standing cultures in 7H9 medium supplemented with OADC (bovine albumin, dextrose, catalase, and Tween-80). Transcript levels were measured from three experimental replicates. In separate experiments, changes in the abundance of several classes of lipids were measured via thin-layer chromatography (TLC). PhoP has been shown to regulate cellular aggregation, growth after macrophage infection, and the production of lipids important for the structure of the cell wall, for virulence, and for the production storage lipids ([25](#_ENREF_25), [85-88](#_ENREF_85)). Using the transcriptomic data, we compared our model predictions with measured lipid changes.

As above, in order to estimate the significance of predicted changes in MFC, we generated a null model distribution by adding simulated gene expression measurement noise to the values from the control channel (see Methods). Predicted changes in MFC were compared to this distribution to calculate a z-score. Predicted MFC values falling outside of the 95% null interval of these MFC values were considered to be significant changes (these values are plotted in Figure S2).

In Figure 2A, we show z-scores of fold changes in predicted maximum flux capacity (MFC) resulting from the *phoP* knockout expression data. We correctly predict changes in the production of 7 measured lipids between the wild type strain and the knockout strain. Our method predicts large increases in the production of the storage lipid triacylglycerol (TAG) and large decreases in the production of virulence lipids sulfolipid (SL-1) and poly- and di-acyltrehaloses (PAT and DATs). The method predicts no changes in either TDMs or trehalose mono-mycolates (TMMs). All these predictions are in qualitative agreement with experimental measurements ([25](#_ENREF_25), [86](#_ENREF_86), [87](#_ENREF_87)).

Of particular interest are the predictions of decreases in SL-1 and PAT, and DAT production and the increase in the production of DIM. PhoP directly regulates *pks2* and *pks3*, genes responsible for the production of sulfolipids ([89](#_ENREF_89)) and polyacyltrehalose/diacyltrehalose (PAT/DAT) ([90](#_ENREF_90)) respectively. PDIM appears to be specifically required for growth in the lungs of mice ([91](#_ENREF_91)) and, along with other transcription factors, plays a role in the regulation of the redox state of the cell by acting as a shunt for the incorporation of reducing equivalents and propionyl-CoA ([92](#_ENREF_92)). It has also been shown that SL-1 and PDIM production can be regulated by the availability of their common precursors methylmalonyl-CoA and propionate ([92](#_ENREF_92)). Our model predictions are consistent with a regulatory role for PhoP in fine-tuning the flux of these precursors to down-stream lipid production pathways ([93](#_ENREF_93)).

We next analyzed the TF DosR. DosR is known to play an important role in the regulation of hypoxic adaptation ([23](#_ENREF_23)). Park and colleagues knocked out the transcription DosR and measured gene expression in both the wild type and knockout strains before and after exposure to hypoxia ([23](#_ENREF_23)) (GSE8829). Using E-Flux-MFC, we analyzed the gene expression data from this experiment. The expression data consists of two sets of two-color microarrays. One set compared gene expression between hypoxia and normoxia for wild type BCG. The other set compared gene expression between hypoxia and normoxia for Δ*dosR*. We used out approach to predict the impact of *dosR* deletion. To do so, we first generated MFC predictions for each lipid class for each condition and strain. We then calculated the fold change in the MFC values between WT and Δ*dosR* in each condition to estimate changes in lipid content. Significance and z-scores were calculated as above (see Methods).

In Figure 2B we plot the predicted changes in selected lipid classes for Δ*dosR* relative to WT in the hypoxic condition. The most significant predicted change is a decrease in TAGs, consistent with previous data. DosR has been shown to directly regulate the TAG production gene *tgs1* (Rv3130c) ([23](#_ENREF_23), [83](#_ENREF_83), [94](#_ENREF_94)). A combination of ChIP-Seq and transcription factor induction experiments further highlighted the strength of regulation of *tgs1* by DosR ([31](#_ENREF_31)). We do not predict significant changes in the production of other classes of lipids. Although substantial changes in other lipids occur during hypoxia (Figure **1**), in this experiment, gene expression measurements were collected after two hours of exposure to hypoxic conditions. Thus, these data are representative of the very early response to hypoxic conditions. DosR, along with Rv0081, appears to play a large role in the initial hypoxic response, after which longer-term adaptations to hypoxic conditions may be the result of regulation by other transcription factors ([24](#_ENREF_24), [31](#_ENREF_31)).

### Predicting changes in lipid expression after the induction of PhoP or DosR

We next sought to simulate the effect on lipid production of the induction of PhoP or DosR. For this, we used previously published measured global expression data for experiments in which each TF was induced via the introduction of an exogenous promoter ([31](#_ENREF_31)). Each of these data sets captures both the direct and indirect effects of TF induction on gene expression. Although experimental measurements of lipid production after induction are not available for either TF, one conceivable outcome is that lipid changes would mirror the results of TF deletion.

This was predicted to be the case for DosR induction, but interestingly not for PhoP induction (Figure 2C and D). For DosR, the most significant prediction of our analysis is an increase in production of TAGs. This is consistent with expectation, given the known regulation of *tgs1* by DosR described above. It is also consistent with the experimentally observed observation that certain strains (W-Beijing lineage) of MTB with constitutively active members of the DosR regulon are associated with overproduction of TAG in aerobic culture ([94](#_ENREF_94)).

We also predict a small but significant decrease in the production of PDIM (Figure 2D). These changes are consistent with decreases in the production of PDIM observed in lipidomics measurements across a hypoxic time course ([31](#_ENREF_31)). Moreover, ChIP-Seq predicts that several genes thought to play a role in PDIM synthesis and transport - Rv2935, Rv2936, and Rv2939 ([95](#_ENREF_95)) - are regulated by DosR ([31](#_ENREF_31)). All three genes are strongly downregulated in the DosR induction dataset. Although the role of *dosR* in the regulation of triacylglycerol is well known, the role of *dosR* in the regulation of PDIM synthesis is less well studied. It has been observed that PDIM concentrations in the cell decrease moderately upon the introduction of hypoxia and then return to normal levels after re-aeration ([31](#_ENREF_31)).

The predicted effects of PhoP induction on lipid production, conversely, did not completely mirror the known effects of *phoP* deletion (Figure 2C). Our analysis predicts an elimination of TAG production and a reversal of SL-1 production, opposite the effect of PhoP deletion. However, PAT, DAT, and TDM are predicted to decrease in their production, as they are in the PhoP deletion ([25](#_ENREF_25), [86](#_ENREF_86), [87](#_ENREF_87)).

It is known that the induction of genes can produce overlapping but not identical phenotypes when compared with gene deletions ([96](#_ENREF_96)). One possible explanation may be the effect of downstream regulatory interactions, gene expression changes, and feedback associated with changes in cellular state. To attempt to partially eliminate these indirect effects, and to attempt to tease out the direct effects from the global effects, we simulated the impact of both PhoP and DosR induction considering expression changes for only those genes predicted to be directly regulated by each TF. We consider a gene to be directly regulated if a strong binding interaction was observed in our previously-generated ChIP-seq data set. For genes not directly regulated by the TF, the mean gene expression values across replicates for corresponding WT samples were used (see Discussion for limitations of this approach).

Figure 2E and Figure 2F show the results for this analysis. The predicted direct effects of DosR induction are qualitatively very similar to the predictions of the global effects (Figure 2F). This predicts that the impact of DosR on changes in these lipids can derive primarily from changes to the direct regulon of DosR. The predicted effects of PhoP induction, however, differ from the predicted global effects for TAG, PAT, and DAT. Induction of only the PhoP regulon is predicted to decrease production of TAGs, mirroring the effect of *phoP* deletion. More surprisingly, PAT and DAT production is predicted to increase, mirroring the effect of PhoP deletion (for DATs) and the global effects of PhoP induction (for PAT and DAT). Changes in PAT and DAT are consistent with the predicted regulation by PhoP of the polyketide synthase *pks3*, known to play a role in the synthesis of acyltrehaloses ([97](#_ENREF_97)). The difference in the direct effects relative to global effects, however, suggests that the impact of this direct regulation is modulated by other indirect changes in cell state.

### Comprehensive prediction of metabolite changes following induction of all MTB TFs.

Bioinformatic analyses suggest that the MTB genome contains roughly 180 transcription factors ([31](#_ENREF_31)). The functions of the majority of these regulators are unknown. To begin to gain insight into the functional roles of unstudied MTB TFs, we have used our approach to predict the potential metabolic impact of the majority of MTB TFs. For this, we have used previously published ([31](#_ENREF_31), [78](#_ENREF_78)) gene expression data for the induction of each MTB TF publicly available at TBDB.org. The gene expression data sets used capture the changes in all genes following TF induction. Using E-Flux-MFC, for each TF we have predicted the metabolic impact on seven major lipid classes (Figure 3A) and all 737 non-currency metabolites (Supplementary Material) in our metabolic model. Predicted changes are quantified as z-scores relative to our background models (see above and Methods), and thus reflect both the significance and magnitude of the predicted impact. TFs functionally annotated in this manner were also clustered to identify sets of regulators with potentially similar functional roles. As in Figure 2E and F, to filter out indirect effects, and thus assess the potential function of the direct regulon of each TF, we also simulated the impact of expression changes for the direct regulon of each TF (Figure 3B and Supplementary Figures). Comparing the predictions for the global effect on lipids in Figure 3A with the predictions of the direct regulon effects in Figure 3B suggests that the majority of TFs may impact lipid production through indirect effects. A similar pattern is seen when examining other metabolites. These data suggest that the full functional significance of a regulator may not be well understood by examining only its directly regulated genes. Instead, the impact of the regulator in the context of the larger regulatory and metabolic network is essential.

# Discussion

We have presented E-Flux-MFC, an enhancement of the original E-Flux method that enables the prediction of changes in the production of both external and internal metabolite corresponding to changes in gene expression data. We validated our method on a genome-scale metabolic model of MTB in two ways. First, we assessed the accuracy of E-Flux-MFC in predicting changes in MTB lipids and metabolites during a time course of hypoxia using previously published metabolomics and transcriptomics data ([31](#_ENREF_31)). Second, we assessed the accuracy of E-Flux-MFC for predicting changes in MTB lipids following the deletion and induction of two well-studied transcription factors. We then use our approach to provide insight into the potential metabolic functions of the majority of MTB TFs, most of which are unstudied. Using our method, we predicted the metabolic impact of the induction of each TF using a previously generated and publically available expression data set ([31](#_ENREF_31), [78](#_ENREF_78)). These predictions provide a resource for studying the large number of TFs whose functions remain unknown, and for identifying TFs that may be associated with metabolites or metabolic pathways of interest.

E-Flux-MFC is an extension of our previously developed method, E-Flux ([46](#_ENREF_46)). Both methods utilize enzyme gene expression data to constrain the maximum flux through the corresponding metabolic reactions at steady state. We and others have previously use E-Flux to accurately predict changes in terminal metabolites ([46](#_ENREF_46), [54](#_ENREF_54)). E-Flux treats the production of such metabolites as an artificial biomass function, and uses FBA with maximum flux constraints to calculate the maximum value of this function. In effect, E-Flux predicts the theoretical maximum flux into a terminal metabolite if the system objective was to solely produce this metabolite. Previous work indicates that changes in this theoretical maximum flux are well correlated with actual changes in metabolite concentrations ([46](#_ENREF_46), [54](#_ENREF_54)).

By focusing on the production of terminal metabolites, E-flux avoids the need to account for the consumption of these metabolites. E-Flux-MFC extends this approach to the prediction of non-terminal metabolites. Net changes in internal metabolites will reflect the balance of production and consumption. This in turn will depend on the activities of enzymes in the corresponding pathways, and the availability of both upstream and downstream metabolites. We hypothesized that gene expression state would provide bounds on the maximum possible production and maximum possible consumption for any given state of internal metabolites. Based on this, E-Flux-MFC uses gene expression data to calculate two intermediate values. First, it combines all the pathways for the production of a target metabolite into a synthetic biomass function, and calculates a theoretical maximum production rate, ignoring consumption. Second, it combines all the pathways for the consumption of a target metabolite into a synthetic biomass function, and calculates a theoretical maximum consumption rate, ignoring production. E-Flux-MFC then calculates the difference between the maximum production flux and the maximum consumption flux in order to calculate a value that we call maximum flux capacity (MFC). MFC represents the theoretical maximum production of a target metabolite if pathways for both production and consumption were operating at their predicted maximums. In additions, while E-Flux applied hard constraints on maximum flux, E-Flux-MFC borrows a key idea from the PROM method ([49](#_ENREF_49)) and allows fluxes that violate the maximum flux constraint, but penalizes such violations.

Several previous methods have addressed the use of gene expression data in order to predict changes in metabolite abundance. Differential producibility analysis (DPA) utilizes FBA to identify genes essential for the production of each metabolite, then utilizes changes in gene expression of essential genes to calculate signals of differential metabolite production ([44](#_ENREF_44)). Reporter metabolite analysis utilizes metabolic network topology to identify metabolites associated with genes that have changed in expression between two conditions ([98](#_ENREF_98)). Reporter feature analysis, a modification of reporter metabolite analysis, has been used to predict metabolites affected by transcription factor perturbations ([99](#_ENREF_99)). Reporter metabolite analysis takes into consideration only those gene expression values directly associated with the reactions that produce and consume a particular metabolite. One of the benefits of our method is that it takes into consideration the fact that the limiting reactions in the production pathway of a particular metabolite may not be the reaction that directly produces a metabolite. The value of the approach taken by DPA is that it utilizes relationships between genes and metabolites that take into account non-direct relationships between genes and the production of specific metabolites. However, neither of these approaches predicts the direction of change in the concentration of a metabolite, one of the main benefits of E-Flux-MFC.

Another method, termed flux imbalance analysis, utilizes an adaptation of the GIMME algorithm ([50](#_ENREF_50)) in order to predict changes in metabolite concentration using gene expression data ([71](#_ENREF_71)). The authors found that their model predictions provide significant predictive value of the sign of the change in a metabolite’s concentration. Although flux imbalance analysis successfully predicts changes in concentration, it utilizes a method that requires the introduction of a required metabolic functionality (RMF), which is a minimal user-defined functionality required for the generation of an expression-constrained flux solution. E-Flux-MFC does not require the definition of an RMF (although one may be enforced if it is well-defined for the condition of interest).

Even if the model accurately predicts the theoretical maximum production and consumption of a metabolite at steady state, changes in these maxima need not result in changes in metabolite levels (if for example production, consumption or both were not operating near the maximal levels). To test the degree to which our predicted MFCs empirically correlate with actual changes in metabolite levels, we performed several validations. First, we assessed the degree to which the method could predict measured changes in MTB metabolites from corresponding measurements of gene expression for the same time points. As shown in Figure 1, the method with our existing genome-scale model of MTB metabolism performs with reasonable accuracy. Predicted changes in MFC for internal metabolites display a statistically significant positive rank and linear correlations (Spearman’s =0.48, p=1.7x10-5, Pearson’s r=0.64, p=1.1x10-9) with measured changes in relative metabolite concentrations (Figure 1B) during the transition from normoxia to hypoxia. In addition, predicted MFCs over the full time course display correspondence with observed changes in lipid concentrations (Figure 1D). Many cell wall lipids might be considered terminal metabolites from the perspective of the current metabolic network model (e.g. phosphatidylethanolamine, for which we are not aware of any recycling reactions in MTB). TAGs, by contrast, are actively produced and consumed by MTB ([80-83](#_ENREF_80)), and the predictions of TAG MFC are in good agreement with measured abundance changes.

Second, we assessed the degree to which the method could predict the metabolic impact of perturbations to transcription factors, given global gene expression data following the perturbation. We studied two TFs – DosR and PhoP – for which such gene expression data are available ([23](#_ENREF_23), [25](#_ENREF_25)), and for which information about expected metabolites changes was also available. For both TFs, E-Flux-MFC is able to correctly predict all known changes (or lack of change) in 7 different lipid classes following TF deletion (Figure 2A and B).

We further examined the predictions of the method based on global gene expression following TF induction ([31](#_ENREF_31)). In this case corresponding lipid measurements do not exist. In these cases, all the results are novel predictions of the model. In some cases, hypotheses could be generated in light of current knowledge to explain the predictions after the fact. In particular, while DosR deletion abolishes TAG production in hypoxia, DosR induction increases TAG production. This is consistent with a report that strains of the W-Beijing lineage of MTB with constitutively active members of the DosR regulon are associated with overproduction of TAG ([94](#_ENREF_94)). Also, the model predicts a weak effect of DosR induction on PDIM. This hypothesis is consistent with the predicted regulation by DosR, based on ChIP-Seq ([31](#_ENREF_31)), of several genes thought to play a role in PDIM synthesis (Rv2935, Rv2936, and Rv2939) ([95](#_ENREF_95)), data that was not used in the modeling. These explanations, however, remain hypotheses generated by the model that require follow-up experimental validation.

The prediction of the impact of PhoP induction was more unexpected. The predictions were complex and did not directly mirror the effects of *phoP* deletion. The known increase in TAG and decrease in SL-1 in Δ*phoP* was either abolished or slightly reversed in the PhoP induction predictions. The decrease in PAT production in Δ*phoP*, however, was enhanced in the predictions for PhoP induction, and DAT was also predicted to decrease in abundance.

Differences in phenotype between gene deletions and gene induction are known to occur ([96](#_ENREF_96)), and this asymmetry would be expected in many cases given the potential complexity and non-linearity of downstream regulatory interactions, gene expression changes, and associated feedback. We explored this by simulating the impact of both PhoP and DosR induction considering expression changes for only those genes predicted to be directly regulated by each TF. For genes not directly regulated by the TF, the WT gene expression values were used. In the case of DosR, both the direct and global effects of induction on the lipids analyzed were similar. The major predicted change was the expected increase in TAG production. For PhoP, conversely, the predicted direct effects on lipid production differed from the predicted global effects. In this case, the predicted direct effects for acyltrehaloses were substantially different from the global prediction, while the impact on TAGs was further magnified. These predictions suggest that the effect of PhoP modulation on lipid production – particularly acyltrehaloses – may depend significantly on the state of other regulators.

We also note that the accuracy of our predictions of changes in lipid production in the PhoP deletion might be enhanced by the use of gene expression data collected from both wild type and Δ*phoP* mutants in acidic conditions, which induce phoP and its regulation of intracellular metabolism via the *aprABC* locus ([25](#_ENREF_25)). Nevertheless, Abramavoitch et al. report a greater than 50 fold reduction in the expression of the *aprABC* locus in the *phoP* transposon mutant, suggesting that even if phoP is not maximally induced in the neutral pH wild type condition, it may still be playing some regulatory role.

Simulating the effect of only the gene expression changes for the direct regulon of a TF has clear limitations. In particular, the synthetic expression state used may not be a realizable state of the system (e.g. if the expression state of the TF regulon imposes hard constraints on the expression of other genes). Moreover, the expression of the genes in a TF regulon may themselves reflect feedback and feedforward effects of the complete network. Such effects would not be filtered out with our approach. Nonetheless, this approach has utility. Predicting the impact of the direct regulation of a TF is conceptually akin to describing the aspects of the function of a TF by considering the functions of the genes in its direct regulon, a common practice. Although the ultimate function of a TF necessarily includes its reciprocal effect on the entire system, the comparison between the global impact and the direct impact can provide insight into what changes depend on interactions of the wider system (e.g. PhoP) and what changes are more independently associated with the direct targets of the TF (e.g. as predicted for TAG and DosR).

Several considerations also impact the interpretation of predictions based on the induction of transcription factors. A primary concern is that induction could result in non-specific changes in gene expression that do not reflect the physiologically relevant function of the induced TF. While this cannot be fully ruled out, we do not observe an overlap in either gene expression or predicted metabolic impact across all the data sets that would imply a specific global effect of the induction protocol. In addition, the metabolic impact of inducing a TF may be an incomplete picture of the function of the TF. Down-regulation or deletion may reveal additional or contrasting effects of the TF. Both are likely important aspects of the TF function. However, to the degree that there is overlap of modulated metabolites, the predictions provide insight even if the directions of the effect may be different between the conditions.

There are several potential reasons for deviations between our model predictions and measured changes in metabolite and lipid abundance. One potential source of error is in our choice of biomass functions. For example, we do not enforce any minimal level of biomass production as with the required metabolic function used in GIMME or TEAM ([50](#_ENREF_50), [71](#_ENREF_71)). Another potential source of error stems from a lack of complete genetic and biochemical knowledge of lipid production pathways. Recent efforts at manual re-annotation ([100](#_ENREF_100)) and condition-specific high-throughput essentiality studies ([38](#_ENREF_38), [101](#_ENREF_101)) have continued to improve the existing model.

We have used our method to provide insight into the functions of transcriptional regulators in MTB. The MTB genome contains roughly 180 transcription factors ([31](#_ENREF_31)), the functions for the majority of which are unknown. Using global gene expression data for the induction of each MTB TF ([31](#_ENREF_31), [78](#_ENREF_78)) publicly available at TBDB.org, we applied our approach to associate each TF with the predicted modulation of 7 major lipid classes (Figure 3) and 207 metabolites (Supplementary Material). Using experimentally determined binding sites derived from ChIP-Seq we have also simulated the metabolic impact of inducing the direct regulons of each TF. The comparison of both simulations provides insight into which functions are mediated by the TF directly, and which may arise as a result of downstream regulatory and metabolic interactions. Comparing results across regulators and metabolites suggests that in the majority of cases metabolites are impacted by TFs through indirect effects. This suggests that the full impact of a regulator can only be understood in the context of the larger regulatory and metabolic network.

# Conclusions

We have presented E-Flux-MFC, an enhancement of the original E-Flux method that enables the prediction of changes in the production of both external and internal metabolite corresponding to changes in gene expression data. We validated our method using multiple datasets combining gene expression and metabolomics measurements. We have used our method to provide insight into the functions of transcriptional regulators in MTB. Using global gene expression data for the induction of each MTB TF we have associated each TF with the potential to modulate each of 7 major lipid classes and 207 metabolites. Using experimentally derived binding sites derived from ChIP-Seq we have also simulated the metabolic impact of inducing the direct regulons of each TF. The comparison of both simulations suggests that in the majority of cases metabolites are impacted by TFs through indirect effects. This indicates that the full impact of a regulator can only be understood in the context of the larger regulatory and metabolic network.

Although we have applied E-Flux-MFC to *Mycobacterium tuberculosis*, it is applicable to any organism for which accurate metabolic models are available. It may also prove useful for both general and tissue-specific models of human metabolism. Several efforts have been undertaken to predict changes in the abundance of metabolic markers in an effort to understand the mechanisms underlying human diseases and to propose novel diagnostics ([102](#_ENREF_102)). The reconstruction of cell-specific models of human metabolism has benefited from the integration of gene expression data collected from those cells. Models describing the metabolism of hepatocytes ([103-105](#_ENREF_103)), macrophages ([47](#_ENREF_47), [106](#_ENREF_106)), and neurons ([107](#_ENREF_107)) have been published, among others.

# Methods

## MTB Metabolic Model

For our analysis, we utilized a modified version of the GSMN-TB model, which was originally described by Beste et al. ([43](#_ENREF_43)). Our modifications were incorporated in order to achieve more agreement with the current state of biochemical knowledge of the pathways responsible for the production of sulfolipid-1, phthiocerol dimycocerosates, triacylglycerol, diacyltrehalose, and polyacyltrehalose. We validated the function of our model by measuring the accuracy of the model for the prediction of gene knockout essentiality. We utilized the transposon site hybridization (TraSH) mutagenesis data set utilized to validate the original GSMN-TB model ([43](#_ENREF_43), [108](#_ENREF_108)). The TraSH data set provides microarray signal ratios that represent the relative abundances of each mutant in the TraSH library. A lower ratio indicates that a particular labeled transposon mutant is present at lower abundance in a culture relative to the abundance of a genomic DNA sample. In order to assign a gene as essential, we apply a threshold to this ratio. Microarray ratios that fall below this threshold are considered to be essential.

For each gene in the data set, we measured the growth rate in the model after the gene had been knocked out. For several different values of the microarray signal ratio, we calculated the area under the curve (AUC) for a receiver-operator characteristic (ROC) curve generated by calculating true positive and false positive rates across a range of growth rate thresholds. We performed this analysis for the original GSMN-TB model and the modified GSMN-TB model at TraSH thresholds of 0.05, 0.1, 0.2, and 0.5. For the original model, we calculate AUCs of 0.72, 0.75, 0.76, and 0.74. For the new model, we calculate AUCs of 0.73, 0.76, 0.77, and 0.73. Thus, the updated model maintains gene knockout prediction accuracy while providing updated representations of important metabolic pathways.

## Transcription factor knockout data

Both of the two-color microarray datasets were analyzed using LIMMA ([109](#_ENREF_109)) and MAANOVA ([110](#_ENREF_110)), microarray analysis libraries for the R statistical programming language ([111](#_ENREF_111)). LIMMA was used to download datasets from LIMMA, for background correction using the normexp model, and for within-array normalization using the print-old

tip loess method. After background correction and normalization, MAANOVA was used as described previously ([46](#_ENREF_46)). We used MAANOVA here to fit an analysis of variance model of the form described in .

As in the model used utilized for analysis of two-color microarray in the E-Flux framework ([46](#_ENREF_46)), denotes the log-transformed measurement from channel , chip , sample , and gene . ­is the value of gene expression that is specific to the sample and gene and is the measurement error. The model is fit to minimize the residual sum of squares. is used as the main input for our metabolic modeling method.

## E-Flux-MFC

In order to answer questions about the accumulation or degradation of both intracellular and extracellular metabolites using the metabolic model of MTB, we developed an extension of the E-Flux and PROM methods called E-Flux-MFC (E-Flux for maximum flux capacity). Both E-Flux and PROM are extensions of a method called flux balance analysis (FBA) ([59](#_ENREF_59)). FBA may be described as the linear programming problem in .

Where is a matrix that captures the stoichiometries of constituent reactions (the stoichiometric matrix), and are vectors describing the upper and lower bounds of each reaction in the model, is the set of fluxes determined by optimizing the objective function, and is an objective function to be maximized and varies depending on the model and the conditions to be simulated.

Generally, these bounds are determined by measuring reaction fluxes through uptake reactions, defining them from physical parameters (e.g. from diffusion constants), or calculating them from thermodynamic constraints ([59](#_ENREF_59)). In both E-Flux and E-Flux-MFC, these bounds are calculated as a function of the expression of the genes that are associated with each reaction. The relationships between genes, proteins, and reactions in the model are represented by Boolean gene-protein-reaction (GPR) formulas.

For some reactions, there is a one-to-one correspondence between genes and the gene product catalyzing that reaction. In these cases, we substitute the gene expression value directly for the reaction expression value. We follow an approach similar to that described in several previous approaches ([46](#_ENREF_46), [68](#_ENREF_68), [72](#_ENREF_72), [75](#_ENREF_75)). In order to utilize these Boolean formulae corresponding to enzyme complexes to calculate a continuous reaction-level expression from gene expression values we incorporate convert AND relationships between genes to the minimum expression value of those two genes. We convert OR relationships the sum of the expressions of two genes. This method handles arbitrarily complex isozyme and enzyme complex relationships. While many factors contribute to enzyme activity, E-Flux-MFC uses gene expression to approximate maximum reaction activity.

In order to ensure that MFC values are comparable between replicates, reaction-level expression values are normalized within each replicate. For each experiment and control pair, we normalize by dividing by the maximum value within each replicate. This calculation yields a value that is not scale-dependent and is thus comparable across replicates. Condition-specific reaction bounds are calculated by multiplying this normalized expression level value by a set of baseline flux bounds determined using flux variability analysis (FVA) after the application of experiment-specific medium constraints, following the approach described Brandes et al. ([68](#_ENREF_68)) using a computationally-efficient implementation ([112](#_ENREF_112)). In FVA, two linear programming problems are solved for each reaction in the model. These problems are described by .

where represents each of reactions in the model, and and are the lower and upper bounds on each reaction flux respectively. Here, is the value of the model objective function and is the minimum value of this objective function to maintain during FVA.

As in PROM, we add a set of constraints on reaction fluxes that are calculated from gene expression data to the original constraints of flux balance analysis. This model is described by the following formulation in .

and are the model bounds and and are the expression-derived flux constraints. minimizes the disagreement between the expression-derived flux bounds and the calculated reaction flux .The relative weighting between the maximization of the objective function (in this case, the maximization of the production or consumption of particular metabolite of interest) is determined by the parameter . The variables and are variables that are chosen by the linear programming solver and that allow the gene expression-weighted upper and lower bounds to be violated in order to provide a more optimal solution (i.e., solutions that are more consistent with the gene expression data). determines the balance between maximizing the value of the objective function and minimizing the sum of the violations of the expression-weighted reaction bounds. Although we constrain our model with both FVA-derived bounds and expression-derived bounds, we have observed that the size of the violation of the expression-derived bounds is generally small relative to the original bounds.

In a process that closely resembles flux variability analysis, we add a reversible demand reaction for each metabolite in turn that allows for us to relax the steady-state assumption for metabolites of interest. By maximizing the flux through the forward and reverse directions of these reactions, we generate values that tell us the maximum production and consumption fluxes for each metabolite in the model. The difference between these maximum production and consumption fluxes is a value that we term the maximum flux capacity (MFC). Between conditions, we calculate fold-changes in MFC by subtracting the experimental value from the control value and dividing by the absolute value of the control value. These fold-change values are converted to z-scores by dividing by the standard deviation of the fold change in MFC across each replicate in an experiment.

## Sampling approach

In analyses utilizing microarray datasets for which replicates were conducted, we utilized expression data values across those replicates to study the effect of variance in gene expression on the final predictions of the model. For each optimization we sample from a Gaussian distribution with mean zero and with a standard deviation calculated from the standard deviation of each gene at each time point across all microarray replicates, utilizing an approach similar to that described in both ([46](#_ENREF_46)) and ([68](#_ENREF_68)). In order to assess the significance of our predictions, we generate samples of gene expression values with this method using the control channel. We generate a null distribution of maximum flux capacities by comparing 1000 sets of control channel samples. We consider a prediction to be significant if it lies outside the interval containing 95% of the control values.

# Availability of Data

The *phoP* knockout data are available in NCBI’s Gene Expression Omnibus (GEO) at accession number GSE22854. The *dosR* knockout and wild type hypoxic transition data are available at GEO accession GSE8829. The hypoxic time course and transcription factor overexpression data are available at GEO accession GSE43466 and on tbdb.org. We provide our complete model as an SBML file in Supplementary Materials. In addition, we have provided in Table S1 the binding network used for the transcription factor overexpression analysis and the median-scaled metabolomics.

# Abbreviations

AUC: area under the curve, ChIP: chromatin immunoprecipitation, DAT: diacyltrehalose, FBA: flux balance analysis, FVA: flux variability analysis, GEO: Gene Expression Omnibus, GIMME: Gene Inactivation Moderated by Metabolism, LIMMA: Linear Models for Microarray Data, MAANOVA: Microarray Analysis of Variance, MDR: multiple drug resistant, MFC: maximum flux capacity, MTB: *Mycobacterium tuberculosis,* NCBI: The National Center for Biotechnology Information, PAT: polyacyltrehalose, PDIM: phthiocerol dimycocerosates, PROM: Probabilistic Regulation of Metabolism, rFBA: regulatory flux balance analysis, ROC: receiver-operator characteristic, SL-1: sulfolipids, TAG: triacylglycerols, TB: tuberculosis, TDM: trehalose dimycolates, TDR: totally drug resistance, TEAM: Temporal Expression-based Analysis of Metabolism, TF: transcription factor, TMM: trehalose monomycolates, XDR: extensively drug resistant

# Acknowledgements

The authors gratefully acknowledge Jeremy Zucker, Matthew Peterson, Elham Azizi and Ed Reznik for help in discussing this project. This work was supported in whole or in part with Federal funds from the National Institute of Allergy and Infectious Diseases National Institute of Health, Department of Health and Human Services, under Contract No. HHSN272200800059C.

# Competing Interests

The authors declare that they have no competing interests.

# Author Contributions

CDG. performed the analysis and wrote the paper. JMD provided advice about metabolic modeling approaches and statistical analysis and proofread the manuscript. JEG conceived and led the project, and wrote the paper. All authors have read and approved the final manuscript.

# References

1. Baddeley A, Dean A, Dias HM, Falzon D, Floyd K, Baena IsG, et al. Global Tuberculosis Report 2014. World Health Organization, 2014.

2. Udwadia Z, Vendoti D. Totally drug-resistant tuberculosis (TDR-TB) in India: every dark cloud has a silver lining. J Epidemiol Community Health. 2013;67(6):471-2.

3. Migliori GB, De Iaco G, Besozzi G, Centis R, Cirillo DM. First tuberculosis cases in Italy resistant to all tested drugs. Euro Surveill. 2007;12(5):E070517 1.

4. Klopper M, Warren RM, Hayes C, Gey van Pittius NC, Streicher EM, Muller B, et al. Emergence and spread of extensively and totally drug-resistant tuberculosis, South Africa. Emerg Infect Dis. 2013;19(3):449-55.

5. Velayati AA, Masjedi MR, Farnia P, Tabarsi P, Ghanavi J, Ziazarifi AH, et al. Emergence of new forms of totally drug-resistant tuberculosis bacilli: super extensively drug-resistant tuberculosis or totally drug-resistant strains in iran. Chest. 2009;136(2):420-5.

6. Chan ED, Iseman MD. Multidrug-resistant and extensively drug-resistant tuberculosis: a review. Curr Opin Infect Dis. 2008;21(6):587-95.

7. Russell DG, Barry CE, 3rd, Flynn JL. Tuberculosis: what we don't know can, and does, hurt us. Science. 2010;328(5980):852-6.

8. Russell DG. Mycobacterium tuberculosis: here today, and here tomorrow. Nat Rev Mol Cell Biol. 2001;2(8):569-77.

9. Russell DG. Who puts the tubercle in tuberculosis? Nat Rev Microbiol. 2006;5(1):39-47.

10. Kaufmann SHE. How can immunology contribute to the control of tuberculosis? Nat Rev Immunol. 2001;1(1):20-30.

11. Carl N. Taming Tuberculosis: A Challenge for Science and Society. Cell Host & Microbe. 2009;5(3):220-4.

12. Dannenberg AM, Jr. Immunopathogenesis of pulmonary tuberculosis. Hosp Pract. 1993;28(1):51-8.

13. Parrish NM, Dick JD, Bishai WR. Mechanisms of latency in *Mycobacterium tuberculosis*. Trends Microbiol. 1998;6(3):107-12.

14. Manabe YC, Bishai WR. Latent *Mycobacterium tuberculosis*-persistence, patience, and winning by waiting. Nat Med. 2000;6(12):1327-9.

15. McKinney JD, Honer zu Bentrup K, Munoz-Elias EJ, Miczak A, Chen B, Chan WT, et al. Persistence of Mycobacterium tuberculosis in macrophages and mice requires the glyoxylate shunt enzyme isocitrate lyase. Nature. 2000;406(6797):735-8.

16. Flynn JL, Chan J. Tuberculosis: latency and reactivation. Infect Immun. 2001;69(7):4195-201.

17. Wayne LG, Sohaskey CD. Nonreplicating persistence of mycobacterium tuberculosis. Annual review of microbiology. 2001;55:139-63.

18. Cosma CL, Sherman DR, Ramakrishnan L. The secret lives of pathogenic mycobacteria. Annu Rev Microbiol. 2003;57:641-76.

19. Gomez JE, McKinney JD. M. tuberculosis persistence, latency, and drug tolerance. Tuberculosis. 2004;84(1-2):29-44.

20. Sherman DR, Voskuil M, Schnappinger D, Liao R, Harrell MI, Schoolnik GK. Regulation of the Mycobacterium tuberculosis hypoxic response gene encoding alpha -crystallin. Proc Natl Acad Sci U S A. 2001;98(13):7534-9.

21. Dasgupta N, Kapur V, Singh KK, Das TK, Sachdeva S, Jyothisri K, et al. Characterization of a two-component system, *devR-devS*, of *Mycobacterium tuberculosis*. Tuber Lung Dis. 2000;80(3):141-59.

22. Boon C, Dick T. *Mycobacterium bovis* response regulator essential for hypoxic dormancy. J Bacteriol. 2002;184(24):6760-7.

23. Park H-D, Guinn KM, Harrell MI, Liao R, Voskuil MI, Tompa M, et al. Rv3133c/dosR is a transcription factor that mediates the hypoxic response of Mycobacterium tuberculosis. Mol Microbiol. 2003;48(3):833-43.

24. Rustad TR, Harrell MI, Liao R, Sherman DR. The enduring hypoxic response of Mycobacterium tuberculosis. PLoS One. 2008;3(1):e1502.

25. Abramovitch RB, Rohde KH, Hsu FF, Russell DG. aprABC: a Mycobacterium tuberculosis complex-specific locus that modulates pH-driven adaptation to the macrophage phagosome. Mol Microbiol. 2011;80(3):678-94.

26. Voskuil MI, Bartek IL, Visconti K, Schoolnik GK. The response of mycobacterium tuberculosis to reactive oxygen and nitrogen species. Frontiers in Microbiology. 2011;2:105.

27. Boshoff HI, Xu X, Tahlan K, Dowd CS, Pethe K, Camacho LR, et al. Biosynthesis and recycling of nicotinamide cofactors in mycobacterium tuberculosis. An essential role for NAD in nonreplicating bacilli. J Biol Chem. 2008;283(28):19329-41.

28. Kumar A, Toledo JC, Patel RP, Lancaster JR, Steyn AJ. Mycobacterium tuberculosis DosS is a redox sensor and DosT is a hypoxia sensor. Proc Natl Acad Sci U S A. 2007;104(28):11568 - 73.

29. Singh A, Crossman DK, Mai D, Guidry L, Voskuil MI, Renfrow MB, et al. Mycobacterium tuberculosis WhiB3 maintains redox homeostasis by regulating virulence lipid anabolism to modulate macrophage response. PLoS Pathog. 2009;5(8):e1000545.

30. Singh A, Guidry L, Narasimhulu KV, Mai D, Trombley J, Redding KE, et al. Mycobacterium tuberculosis WhiB3 responds to O2 and nitric oxide via its [4Fe-4S] cluster and is essential for nutrient starvation survival. Proc Natl Acad Sci U S A. 2007;104(28):11562-7.

31. Galagan JE, Minch K, Peterson M, Lyubetskaya A, Azizi E, Sweet L, et al. The Mycobacterium tuberculosis regulatory network and hypoxia. Nature. 2013;499(7457):178-83.

32. Schnappinger D, Ehrt S, Voskuil MI, Liu Y, Mangan JA, Monahan IM, et al. Transcriptional Adaptation of Mycobacterium tuberculosis within Macrophages: Insights into the Phagosomal Environment. J Exp Med. 2003;198(5):693-704.

33. Yang X, Nesbitt NM, Dubnau E, Smith I, Sampson NS. Cholesterol metabolism increases the metabolic pool of propionate in Mycobacterium tuberculosis. Biochemistry. 2009;48(18):3819-21.

34. Miner MD, Chang JC, Pandey AK, Sassetti CM, Sherman DR. Role of cholesterol in Mycobacterium tuberculosis infection. Indian J Exp Biol. 2009;47(6):407-11.

35. Chang JC, Miner MD, Pandey AK, Gill WP, Harik NS, Sassetti CM, et al. igr Genes and Mycobacterium tuberculosis cholesterol metabolism. J Bacteriol. 2009;191(16):5232-9.

36. Kendall SL, Burgess P, Balhana R, Withers M, Ten Bokum A, Lott JS, et al. Cholesterol utilization in mycobacteria is controlled by two TetR-type transcriptional regulators: kstR and kstR2. Microbiology. 2010;156(Pt 5):1362-71.

37. Kendall SL, Withers M, Soffair CN, Moreland NJ, Gurcha S, Sidders B, et al. A highly conserved transcriptional repressor controls a large regulon involved in lipid degradation in Mycobacterium smegmatis and Mycobacterium tuberculosis. Mol Microbiol. 2007;65(3):684-99.

38. Nesbitt NM, Yang X, Fontan P, Kolesnikova I, Smith I, Sampson NS, et al. A thiolase of Mycobacterium tuberculosis is required for virulence and production of androstenedione and androstadienedione from cholesterol. Infect Immun. 2010;78(1):275-82.

39. Uhia I, Galan B, Medrano FJ, Garcia JL. Characterization of the KstR-dependent promoter of the gene for the first step of the cholesterol degradative pathway in Mycobacterium smegmatis. Microbiology. 2011;157(Pt 9):2670-80.

40. Russell DG, VanderVen BC, Lee W, Abramovitch RB, Kim MJ, Homolka S, et al. Mycobacterium tuberculosis wears what it eats. Cell Host Microbe. 2010;8(1):68-76.

41. Russell DG, Mwandumba HC, Rhoades EE. Mycobacterium and the coat of many lipids. J Cell Biol. 2002;158(3):421-6.

42. Jamshidi N, Palsson BO. Investigating the metabolic capabilities of Mycobacterium tuberculosis H37Rv using the in silico strain iNJ661 and proposing alternative drug targets. BMC Syst Biol. 2007;1:26.

43. Beste DJ, Hooper T, Stewart G, Bonde B, Avignone-Rossa C, Bushell ME, et al. GSMN-TB: a web-based genome-scale network model of Mycobacterium tuberculosis metabolism. Genome Biol. 2007;8(5):R89.

44. Bonde BK, Beste DJV, Laing E, Kierzek AM, McFadden J. Differential producibility analysis (DPA) of transcriptomic data with metabolic networks: deconstructing the metabolic response of M. tuberculosis. PLoS Comput Biol. 2011;7(6):e1002060.

45. Shi L, Sohaskey CD, Pfeiffer C, Datta P, Parks M, McFadden J, et al. Carbon flux rerouting during Mycobacterium tuberculosis growth arrest. Mol Microbiol. 2010;78(5):1199-215.

46. Colijn C, Brandes A, Zucker J, Lun DS, Weiner B, Farhat MR, et al. Interpreting expression data with metabolic flux models: predicting Mycobacterium tuberculosis mycolic acid production. PLoS Comput Biol. 2009;5(8):e1000489.

47. Bordbar A, Lewis NE, Schellenberger J, Palsson BO, Jamshidi N. Insight into human alveolar macrophage and M. tuberculosis interactions via metabolic reconstructions. Mol Syst Biol. 2010;6.

48. Chindelevitch L, Stanley S, Hung D, Regev A, Berger B. MetaMerge: scaling up genome-scale metabolic reconstructions with application to Mycobacterium tuberculosis. Genome Biol. 2012;13(1):r6.

49. Chandrasekaran S, Price ND. Probabilistic integrative modeling of genome-scale metabolic and regulatory networks in Escherichia coli and Mycobacterium tuberculosis. Proc Natl Acad Sci U S A. 2010;107(41):17845-50.

50. Becker SA, Palsson BO. Context-specific metabolic networks are consistent with experiments. PLoS Comput Biol. 2008;4(5):e1000082.

51. Lewis NE, Hixson KK, Conrad TM, Lerman JA, Charusanti P, Polpitiya AD, et al. Omic data from evolved E. coli are consistent with computed optimal growth from genome-scale models. Mol Syst Biol. 2010;6.

52. Raghunathan A, Reed J, Shin S, Palsson B, Daefler S. Constraint-based analysis of metabolic capacity of Salmonella typhimurium during host-pathogen interaction. BMC Syst Biol. 2009;3(1):38-.

53. Huthmacher C, Hoppe A, Bulik S, Holzhutter HG. Antimalarial drug targets in Plasmodium falciparum predicted by stage-specific metabolic network analysis. BMC Syst Biol. 2010;4(1):120.

54. Plata G, Hsiao TL, Olszewski KL, Llinas M, Vitkup D. Reconstruction and flux-balance analysis of the Plasmodium falciparum metabolic network. Mol Syst Biol. 2010;6:408.

55. Ma H, Sorokin A, Mazein A, Selkov A, Selkov E, Demin O, et al. The Edinburgh human metabolic network reconstruction and its functional analysis. Mol Syst Biol. 2007;3(1):n/a-n/a.

56. Duarte NC, Becker SA, Jamshidi N, Thiele I, Mo ML, Vo TD, et al. Global reconstruction of the human metabolic network based on genomic and bibliomic data. Proceedings of the National Academy of Sciences. 2007;104(6):1777-82.

57. Lofthouse EK, Wheeler PR, Beste DJ, Khatri BL, Wu H, Mendum TA, et al. Systems-based approaches to probing metabolic variation within the Mycobacterium tuberculosis complex. PLoS One. 2013;8(9):e75913.

58. Fang X, Wallqvist A, Reifman J. Development and analysis of an in vivo-compatible metabolic network of Mycobacterium tuberculosis. BMC Syst Biol. 2010;4:160.

59. Orth JD, Thiele I, Palsson BO. What is flux balance analysis? Nat Biotechnol. 2010;28(3):245-8.

60. Papin JA, Price ND, Wiback SJ, Fell DA, Palsson BO. Metabolic pathways in the post-genome era. Trends Biochem Sci. 2003;28(5):250-8.

61. Schilling CH, Edwards JS, Palsson BO. Toward metabolic phenomics: analysis of genomic data using flux balances. Biotechnol Prog. 1999;15(3):288-95.

62. Schilling CH, Schuster S, Palsson BO, Heinrich R. Metabolic pathway analysis: basic concepts and scientific applications in the post-genomic era. Biotechnol Prog. 1999;15(3):296-303.

63. Varma A, Palsson BO. Stoichiometric flux balance models quantitatively predict growth and metabolic by-product secretion in wild-type Escherichia coli W3110. Appl Environ Microbiol. 1994;60(10):3724-31.

64. Wagner C, Urbanczik R. The geometry of the flux cone of a metabolic network. Biophys J. 2005;89(6):3837-45.

65. Edwards JS, Palsson BO. Metabolic flux balance analysis and the in silico analysis of Escherichia coli K-12 gene deletions. BMC Bioinformatics. 2000;1:1.

66. Famili I, Forster J, Nielsen J, Palsson BO. Saccharomyces cerevisiae phenotypes can be predicted by using constraint-based analysis of a genome-scale reconstructed metabolic network. Proc Natl Acad Sci U S A. 2003;100(23):13134-9.

67. Fong SS, Palsson BO. Metabolic gene-deletion strains of Escherichia coli evolve to computationally predicted growth phenotypes. Nat Genet. 2004;36(10):1056-8.

68. Brandes A, Lun DS, Ip K, Zucker J, Colijn C, Weiner B, et al. Inferring carbon sources from gene expression profiles using metabolic flux models. PLoS One. 2012;7(5):e36947.

69. Edwards JS, Ibarra RU, Palsson BO. In silico predictions of Escherichia coli metabolic capabilities are consistent with experimental data. Nat Biotechnol. 2001;19(2):125-30.

70. Dreyfuss JM, Zucker JD, Hood HM, Ocasio LR, Sachs MS, Galagan JE. Reconstruction and validation of a genome-scale metabolic model for the filamentous fungus Neurospora crassa using FARM. PLoS Comput Biol. 2013;9(7):e1003126.

71. Reznik E, Mehta P, Segre D. Flux imbalance analysis and the sensitivity of cellular growth to changes in metabolite pools. PLoS Comput Biol. 2013;9(8):e1003195.

72. Collins SB, Reznik E, Segre D. Temporal expression-based analysis of metabolism. PLoS Comput Biol. 2012;8(11):e1002781.

73. Lewis NE, Nagarajan H, Palsson BO. Constraining the metabolic genotype-phenotype relationship using a phylogeny of in silico methods. Nat Rev Microbiol. 2012;10(4):291-305.

74. Covert MW, Schilling CH, Palsson B. Regulation of Gene Expression in Flux Balance Models of Metabolism. J Theor Biol. 2001;213(1):73-88.

75. Shlomi T, Cabili MN, Herrgard MJ, Palsson BO, Ruppin E. Network-based prediction of human tissue-specific metabolism. Nat Biotechnol. 2008;26(9):1003-10.

76. Zur H, Ruppin E, Shlomi T. iMAT: an integrative metabolic analysis tool. Bioinformatics. 2010;26(24):3140-2.

77. Boshoff HI, Myers TG, Copp BR, McNeil MR, Wilson MA, Barry CE, 3rd. The transcriptional responses of Mycobacterium tuberculosis to inhibitors of metabolism: novel insights into drug mechanisms of action. J Biol Chem. 2004;279(38):40174-84.

78. Rustad TR, Minch KJ, Ma S, Winkler JK, Hobbs S, Hickey M, et al. Mapping and manipulating the Mycobacterium tuberculosis transcriptome using a transcription factor overexpression-derived regulatory network. Genome biology. 2014;15(11):502.

79. Mahadevan R, Edwards JS, Doyle FJ, 3rd. Dynamic flux balance analysis of diauxic growth in Escherichia coli. Biophys J. 2002;83(3):1331-40.

80. Daniel J, Maamar H, Deb C, Sirakova TD, Kolattukudy PE. Mycobacterium tuberculosis uses host triacylglycerol to accumulate lipid droplets and acquires a dormancy-like phenotype in lipid-loaded macrophages. PLoS Pathog. 2011;7(6):e1002093.

81. Deb C, Daniel J, Sirakova TD, Abomoelak B, Dubey VS, Kolattukudy PE. A novel lipase belonging to the hormone-sensitive lipase family induced under starvation to utilize stored triacylglycerol in Mycobacterium tuberculosis. The Journal of biological chemistry. 2006;281(7):3866-75.

82. Low KL, Rao PS, Shui G, Bendt AK, Pethe K, Dick T, et al. Triacylglycerol utilization is required for regrowth of in vitro hypoxic nonreplicating Mycobacterium bovis bacillus Calmette-Guerin. J Bacteriol. 2009;191(16):5037-43.

83. Daniel J, Deb C, Dubey VS, Sirakova TD, Abomoelak B, Morbidoni HR, et al. Induction of a novel class of diacylglycerol acyltransferases and triacylglycerol accumulation in Mycobacterium tuberculosis as it goes into a dormancy-like state in culture. J Bacteriol. 2004;186(15):5017-30.

84. Baek SH, Li AH, Sassetti CM. Metabolic regulation of mycobacterial growth and antibiotic sensitivity. PLoS Biol. 2011;9(5):e1001065.

85. Pérez E, Samper S, Bordas Y, Guilhot C, Gicquel B, Martín C. An essential role for phoP in Mycobacterium tuberculosis virulence. Mol Microbiol. 2001;41(1):179-87.

86. Walters SB, Dubnau E, Kolesnikova I, Laval F, Daffe M, Smith I. The Mycobacterium tuberculosis PhoPR two-component system regulates genes essential for virulence and complex lipid biosynthesis. Mol Microbiol. 2006;60(2):312-30.

87. Gonzalo Asensio J, Maia C, Ferrer NL, Barilone N, Laval F, Soto CY, et al. The virulence-associated two-component PhoP-PhoR system controls the biosynthesis of polyketide-derived lipids in Mycobacterium tuberculosis. J Biol Chem. 2006;281(3):1313-6.

88. Gonzalo-Asensio J, Malaga W, Pawlik A, Astarie-Dequeker C, Passemar C, Moreau F, et al. Evolutionary history of tuberculosis shaped by conserved mutations in the PhoPR virulence regulator. Proceedings of the National Academy of Sciences. 2014;111(31):11491-6.

89. Sirakova TD, Thirumala AK, Dubey VS, Sprecher H, Kolattukudy PE. The Mycobacterium tuberculosis pks2 Gene Encodes the Synthase for the Hepta- and Octamethyl-branched Fatty Acids Required for Sulfolipid Synthesis. J Biol Chem. 2001;276(20):16833-9.

90. Jackson JGA, Catarina M, Nadia LF, Nathalie B, Françoise L, Carlos Yesid S, et al. The Virulence-associated Two-component PhoP-PhoR System Controls the Biosynthesis of Polyketide-derived Lipids in Mycobacterium tuberculosis. 2006.

91. Cox JS, Chen B, McNeil M, Jacobs WR, Jr. Complex lipid determines tissue-specific replication of Mycobacterium tuberculosis in mice. Nature. 1999;402(6757):79-83.

92. Jain M, Petzold CJ, Schelle MW, Leavell MD, Mougous JD, Bertozzi CR, et al. Lipidomics reveals control of Mycobacterium tuberculosis virulence lipids via metabolic coupling. Proc Natl Acad Sci U S A. 2007;104(12):5133-8.

93. Russell WL, Brian CV, Ruth JF, David G. Intracellular Mycobacterium tuberculosis Exploits Host-derived Fatty Acids to Limit Metabolic Stress. J Biol Chem. 2013;288(10):6788-800.

94. Reed MB, Gagneux S, Deriemer K, Small PM, Barry CE, 3rd. The W-Beijing lineage of Mycobacterium tuberculosis overproduces triglycerides and has the DosR dormancy regulon constitutively upregulated. J Bacteriol. 2007;189(7):2583-9.

95. Buglino J, Onwueme KC, Ferreras JA, Quadri LE, Lima CD. Crystal structure of PapA5, a phthiocerol dimycocerosyl transferase from Mycobacterium tuberculosis. J Biol Chem. 2004;279(29):30634-42.

96. Sopko R, Huang D, Preston N, Chua G, Papp B, Kafadar K, et al. Mapping Pathways and Phenotypes by Systematic Gene Overexpression. Mol Cell. 2006;21(3):319-30.

97. Dubey VS, Sirakova TD, Kolattukudy PE. Disruption of msl3 abolishes the synthesis of mycolipanoic and mycolipenic acids required for polyacyltrehalose synthesis in Mycobacterium tuberculosis H37Rv and causes cell aggregation. Mol Microbiol. 2002;45(5):1451-9.

98. Patil KR, Nielsen J. Uncovering transcriptional regulation of metabolism by using metabolic network topology. Proc Natl Acad Sci U S A. 2005;102(8):2685-9.

99. Oliveira AP, Patil KR, Nielsen J. Architecture of transcriptional regulatory circuits is knitted over the topology of bio-molecular interaction networks. BMC Syst Biol. 2008;2:17.

100. Slayden RA, Jackson M, Zucker J, Ramirez MV, Dawson CC, Crew R, et al. Updating and curating metabolic pathways of TB. Tuberculosis. 2013;93(1):47-59.

101. Griffin JE, Pandey AK, Gilmore SA, Mizrahi V, McKinney JD, Bertozzi CR, et al. Cholesterol catabolism by Mycobacterium tuberculosis requires transcriptional and metabolic adaptations. Chem Biol. 2012;19(2):218-27.

102. Shlomi T, Cabili MN, Ruppin E. Predicting metabolic biomarkers of human inborn errors of metabolism. Mol Syst Biol. 2009;5:263-.

103. Jerby L, Shlomi T, Ruppin E. Computational reconstruction of tissue‐specific metabolic models: application to human liver metabolism2010 2010-01-01 00:00:00.

104. Folger O, Jerby L, Frezza C, Gottlieb E, Ruppin E, Shlomi T. Predicting selective drug targets in cancer through metabolic networks2011.

105. Gille C, Bölling C, Hoppe A, Bulik S, Hoffmann S, Hübner K, et al. HepatoNet1: a comprehensive metabolic reconstruction of the human hepatocyte for the analysis of liver physiology. Mol Syst Biol. 2010;6(1).

106. Bordbar A, Mo ML, Nakayasu ES, Schrimpe‐Rutledge AC, Kim YM, Metz TO, et al. Model‐driven multi‐omic data analysis elucidates metabolic immunomodulators of macrophage activation2012.

107. Lewis NE, Schramm G, Bordbar A, Schellenberger J, Andersen MP, Cheng JK, et al. Large-scale in silico modeling of metabolic interactions between cell types in the human brain. Nat Biotechnol. 2010;28(12):1279-85.

108. Sassetti CM, Boyd DH, Rubin EJ. Genes required for mycobacterial growth defined by high density mutagenesis. Mol Microbiol. 2003;48(1):77-84.

109. Smyth GK. Linear models and empirical bayes methods for assessing differential expression in microarray experiments. Stat Appl Genet Mol Biol. 2004;3:Article3.

110. Wu H, Kerr MK, Cui X, Churchill GA. MAANOVA: a software package for the analysis of spotted cDNA microarray experiments. The analysis of gene expression data: methods and software. 2003:313-41.

111. R Development Core Team. R: A Language and Environment for Statistical Computing: R Foundation for Statistical Computing; 2011. Available from: <http://www.R-project.org>.

112. Gudmundsson S, Thiele I. Computationally efficient flux variability analysis. BMC Bioinformatics. 2010;11:489.

# Figure Legends

Figure 1: Validation of prediction of changes in MTB metabolite production during changes in oxygen. (A) The predicted change in maximum flux through each metabolite with a corresponding metabolomics measurement is plotted against the log2 fold change in concentration from just prior to hypoxia and 1-day post hypoxia. Error bars represent standard deviations of predicted changes calculated across the 1000 samples in the case of model predictions and across 4 replicates in the case of experimental measurements. MFC values for all model metabolites are provided in Figure S1 in Supplementary Materials. (B) 1000 samples were generated by randomizing gene labels on the time course expression data. The Spearman correlation coefficient was calculated for each permutation. This distribution is compared with the distribution of coefficients generated from Monte Carlo samples using the correct gene labels. (C) Spearman correlation coefficient as a function of the parameter for predictions of change in metabolite concentration from just prior to hypoxia to 1-day post hypoxia. Error bars represent standard deviations of the Spearman correlation coefficient calculated across 100 samples of the gene expression data. (D) Changes in the production of several classes of lipids across the full hypoxic and reaeration time course. Red lines show model predictions of normalized net production for each lipid across the experimental time course. Black lines show normalized measured changed in abundance across each time course for each measured member of the lipid class. Error bars represent the standard deviation across samples for predicted production and across experimental replicates for measured abundance values. (E) Predicted changes in TAG production (red) and consumption (blue) fluxes. Error bars represent the standard deviation across samples for predicted production and consumption.

Figure 2: Predicted changes in lipid production are deletion or induction of *phoP* and *dosR*. (A) Predicted changes in lipid production in a *phoP* knockout strain. E-Flux-MFC correctly predicts the change in production of 7/7 previously-measured changes in lipid production in *phoP* knockout mutants ([25](#_ENREF_25), [86](#_ENREF_86), [87](#_ENREF_87)). (B) Predicted changes in lipid production in a *dosR* knockout strain after the induction of hypoxia (2 hours 0.2% O2) ([23](#_ENREF_23)). TAG production significantly increased, in agreement with expectations based on previous observations. (C) Predicted changes in lipid production after the induction of *phoP*. (D) Predicted changes in lipid production after the induction of *dosR*. (E) Predictions of changes in lipid production specific to the direct regulon of *phoP* after induction. Direct regulon from ChIP-Seq data ([31](#_ENREF_31)). (F) Predicted changes in lipid production specific to the direct regulon of DosR after induction. Abbreviations: TAG (triacylglycerols), PDIM (phthiocerol dimycocerosates), SL-1 (sulfolipids), PAT (polyacyltrehalose), DAT (diacyltrehalose), TDM (trehalose dimycolates), TMM (trehalose monomycolates).

Figure 3: Predicted impact of the induction of 207 TFs on 7 lipid classes. (A) Predictions based on global gene expression after TF induction. Left panel displays results for all TFs clustered by similarity in metabolite profile. Right panels display individual clusters of TFs. Red indicates that TF induction is predicted to increase metabolite production while blue indicates decreased predicted production. (B) Predictions based on expression of the direct regulon of each TF after TF induction. The expression of other genes is set to the mean expression in wild type induction control experiments.

Figure S1: Distribution of maximum flux capacities for all model metabolites. Histogram of MFC values for all model metabolites. 40% of the metabolites in our model (305/754) have an MFC of zero. 300 of these are neither produced nor consumed in our model, likely due to medium constraints placed on the model. External hydrogen is not plotted due its large MFC (approximately 48.4).

# Supporting Information

Supplementary Data Files:

- Pathways.zip - full set model predictions for all metabolites during transcription factor induction based on genome-wise expression
- Pathways_specific.zip - full set model predictions for all metabolites during transcription factor induction based on TF regulon specific expression
- Model.xml: Genome scale MTB metabolic model used
- Table_S1.xlsx - the binding network used for the transcription factor overexpression analyses and median-scaled metabolite abundance values for normoxic and hypoxic conditions
